# Tumor microenvironment heterogeneity an important mediator of prostate cancer progression and therapeutic resistance

**DOI:** 10.1038/s41698-022-00272-w

**Published:** 2022-05-04

**Authors:** Rongbin Ge, Zongwei Wang, Liang Cheng

**Affiliations:** 1 Department of Pathology and Laboratory Medicine, Indiana University School of Medicine, Indianapolis, IN, USA; 2 Department of Surgery, Division of Urologic Surgery, Beth Israel Deaconess Medical Center, Harvard Medical School, Boston, MA USA; 3 Department of Urology, Indiana University School of Medicine, Indianapolis, IN, USA

**Keywords:** Urological cancer, Prostate cancer

## Abstract

Prostate cancer is characterized by a high degree of heterogeneity, which poses a major challenge to precision therapy and drug development. In this review, we discuss how nongenetic factors contribute to heterogeneity of prostate cancer. We also discuss tumor heterogeneity and phenotypic switching related to anticancer therapies. Lastly, we summarize the challenges targeting the tumor environments, and emphasize that continued exploration of tumor heterogeneity is needed in order to offer a personalized therapy for advanced prostate cancer patients.

## Introduction

Tumor heterogeneity has been a major contributor to lethal outcomes, drug resistance, and therapeutic failures, and presents a key challenge to precision medicine goals^1,2^. In light of the association between tumor heterogeneity and poor prognostic results, a measure of heterogeneity itself might be useful as a prognostic marker^3^. Although heterogeneity of prostate cancer is conventionally attributed to genetic diversity^4–6^, current evidence reveals that in addition to genetic factors, the tumor heterogeneity could be derived from nongenetic variabilities^7^.

The stroma serves as a main barrier preventing carcinogenesis in benign tissue; however, the presence of cancer cells initiates crucial changes, converting the environment into one that supports tumor growth. These changes include fibroblast recruitment, immunocytes migration, matrix remodeling, development of tumor-specific vasculature, and aberrant epigenetic landscape, and each of these changes might promote heterogeneity of tumor microenvironment (TME). Local diversity of selective pressures in TME, such as hypoxia, acidity, and growth factors, also actively shape tumor morphology. Conceivably, the distinct environmental landscape in the tumor plays a significant role in tumor heterogeneity.

TME is dynamic with spatial and temporal changes in composition in response to environmental pressures and anticancer therapies, and the continued crosstalk between tumor cells and the surrounding microenvironment is fundamental to tumor initiation, phenotypic changes, cancer progression, and therapeutic resistance. In this review, we summarize the challenges targeting the tumor environments, and emphasize that understanding nongenetic mechanisms might open novel diagnostic and therapeutic approaches with the potential to improve the efficacy of current prostate cancer treatments.

### Clonal heterogeneity

**Fig. 1:**
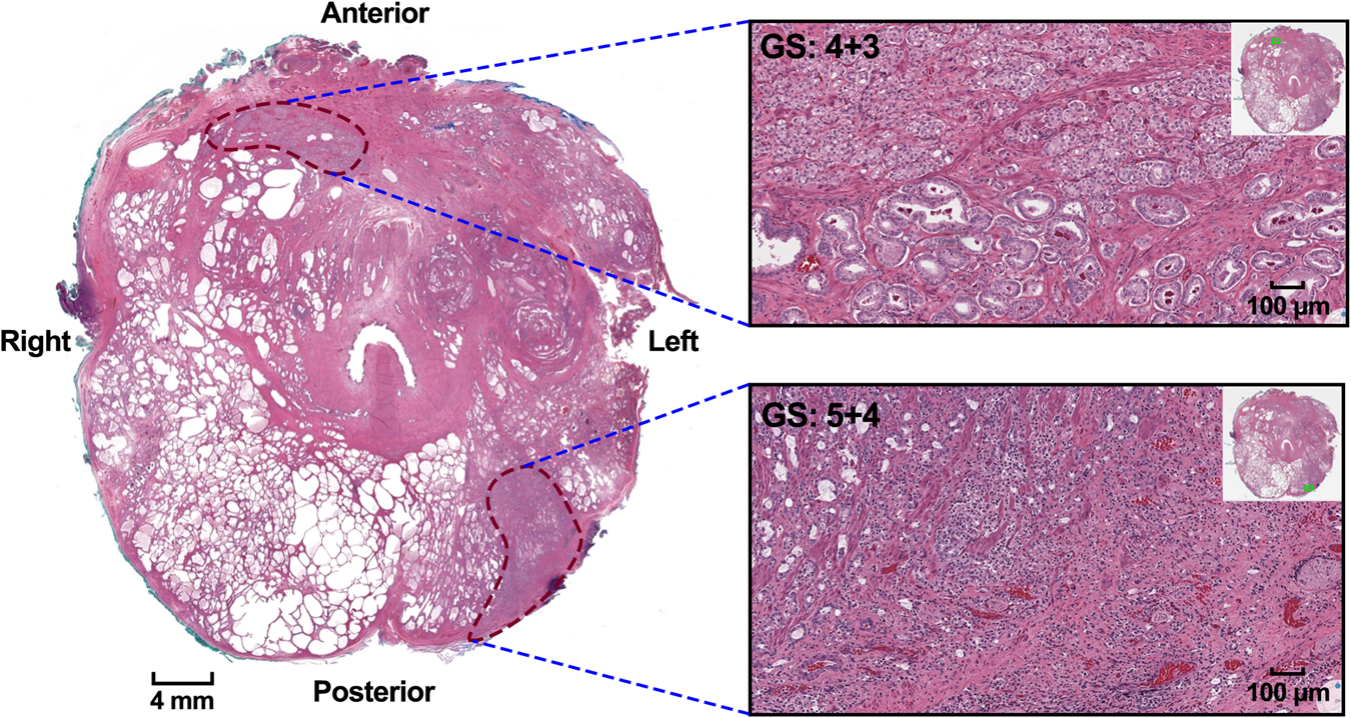
Intratumor and intertumor heterogeneity of prostate cancer. Whole-mount cross-section of a radical prostatectomy specimen has two separated tumor foci. One focus is in the right anterior of the prostate (Gleason score 4 + 3 = 7, Group 3) whereas another focus is in the left posterior of the prostate (Gleason score 5 + 4 = 9, Group 5). Scale bars, 4 mm (left) and 100 µm (right). Methods: Radical prostatectomy specimens were serially sectioned into 3 mm slices and completely embedded. The case was reviewed by a single urologic pathologist (R.G.) in 2021. The following features were monitored: Gleason Score and Grade Group according to the International Society of Urological Pathology (ISUP) 2014 guidelines. The percentages of Gleason pattern 3, 4, and 5 were estimated, including presence of tertiary Gleason patterns.

Prostate cancer is a multifocal disease (Fig. 1) and each focus might have a different phenotype (intertumor heterogeneity)^8^. As the individual tumor volume increases over time, multiple foci might merge into a larger mass exhibiting greater tumor heterogeneity (intratumor heterogeneity). It is controversial whether those separate foci reflect a monoclonal origin^9–12^ or a polyclonal origin^13^. The studies supporting the monoclonal origin argue that both genetic and epigenetic events occur in a single ancestral cell that does not possess all the necessary mutations to transform into a cancer cell. As those cells are exposed to additional events and divide, DNA replication errors lead to daughter cells that are genetically different from each other. Hence, although the cells are heterogeneous, all derive from the same ancestor^14^. For instance, analysis of the whole exome sequencing and transcriptome profiles from Gleason 3 and neighboring Gleason 4 tumor foci revealed that the adjacent tumors emerged early from a common precursor and subsequently undergo independent evolution^10^. In a separate study, analysis of genomewide single nucleotide polymorphism and copy number variations from metastasized prostate cancers demonstrated that most cancers were of monoclonal origin and have identical copy number changes^11^.

In support of the polyclonal tumor origin, the chromosomal analysis and genomic DNA sequencing studies on multifocal prostate cancers have revealed that different tumor foci have independent clonal expansions^5,13,15^. In another study, analysis of the whole exome sequencing of 23 distinct tumor foci from 5 prostate cancer cases demonstrated that the multifocal tumors were highly heterogeneous for single nucleotide variants, copy number aberrations, and genomic rearrangements^16^. Similarly, a large cohort study of 89 tumor foci from 41 different patients revealed that samples from different tumor foci in the same prostatectomy specimen rarely had any shared point mutations and the same DNA copy number changes^8^.

Moreover, analysis of 17 tumor cells from localized lesions with different Gleason scores from 2 prostatectomy cases revealed that in patient number 1, every cell had the same *TP53* mutation, which is consistent with the monoclonal model. In the patient number 2, only one cell subpopulation contained the *TP53* mutation, while other cells carried different mutations, supporting a polyclonal model^17^. Overall, these studies indicated that the origin of prostate cancers may have a monoclonal or polyclonal origin that varies from case to case. Further research may shed more light on the generality or predominance of any of these theories.

### Tumor microenvironment heterogeneity

**Fig. 2:**
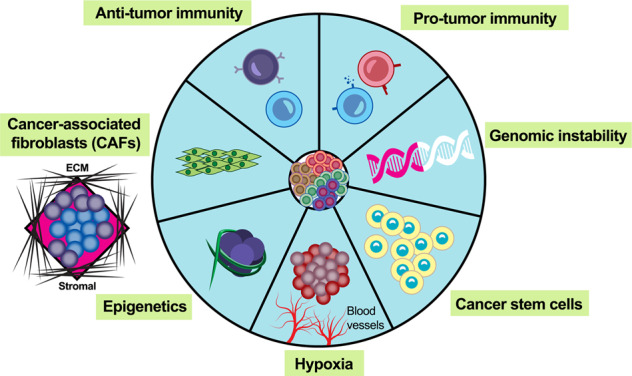
The heterogenous tumor microenvironment. The tumor microenvironment possesses a dynamic topography within the tumor and is composed of cancer-associated fibroblasts, stromal cells, extracellular matrix, and immune cells. Hypoxic status, vasculature, and epigenetics, all may have a complex crosstalk with prostate cancer cells in dynamic ways.

It is well-accepted that tumorigenesis is not only dependent on genetic alterations or epigenetic modifications in cancer cells, but is also regulated by the TME^18^. The TME is composed of fibroblasts, pericytes, immunocytes, and endotheliocytes; each able to crosstalk with cancer cells in dynamic ways (Fig. 2). Usually, the orchestrated impact of microenvironmental components on cancer cells is characterized by the different region, and the tumorigenesis is modulated by the regional heterogeneity in the hypoxia, acidity, and cytokines in a tumor environment^19,20^. Moreover, the cancer-associated fibroblasts (CAFs) are among the most abundant constituents in the TME, contributing to the malignant phenotype at all levels^21–25^. CAFs comprise heterogeneous clusters exerting distinct functions, such as tumor growth, angiogenic process and stromal remodeling, drug resistance, and tumor metastasis^26^. The CAF clusters have either tumor suppressive effects^27,28^ or tumor promoting effects^29,30^. This heterogeneity might result from numerous causes, such as dynamic interaction between tumor cells and stromal cells, extracellular matrix, and cytokines and growth factors secreted into the TME^31^.

Heterogeneity of CAFs is attributed, at least partially, to multiple origins. CAFs are traditionally considered to originate from the resident fibroblasts under the influence of the transforming growth factor (TGF-β) produced by stromal cells and cancer cells^32^ or hypoxia inducible factor (HIF)-1α pathway^22^. The CAF-produced TGF-β and CXCL-12 maintain the myofibroblast phenotype and promote the interaction between tumor-stroma^33,34^. Evidence has suggested that CAFs also derive from an endothelial-mesenchymal or epithelial-mesenchymal transition^35^ which is driven by TGF-β or SMAD signaling^36,37^. Other mature cells, including pericytes or inflammatory cells in stroma, may also transdifferentiate into CAFs through TGF-β modulated induction of mesenchymal-to-mesenchymal transition^38^. Another perspective is that bone marrow-derived mesenchymal stromal cells may differentiate into CAFs^39^. The recruitment of mesenchymal stromal cells and their activation into CAFs are facilitated by TGF-β and CXCL-12 secreted by cancer cells^40,41^. Lastly, cancer stem cells are found to be one of the key origins of CAFs. The transformation of cancer stem cells into stromal cells adds a new dimension that explains tumor heterogeneity^42^. Overall, the multiple origins contribute to the heterogeneity of CAFs.

### Functional heterogeneity of cancer-associated fibroblasts

Functional diversity of prostate CAFs has been observed in some stromal specific markers. For instance, p62 plays a role in the regulation of prostate TME^43^. Loss of p62 in the stroma promotes prostate cancer growth and is associated with high Gleason score tumors^43^. CD90 is another stromal specific marker that is related to cancer progression. Stromal cells with high levels of CD90 have more tumor promoting effects than cells with low levels of CD90^44^. In addition, some stromal biomarkers are related to the cancer phenotypic switch. For example, CXCL-13 is expressed in tumor-associated myofibroblasts and induced by depleted androgen. The rise of CXCL-13 is related to the progress of advanced prostate cancer^45^. Likewise, CD105, the cell surface endoglin, is heterogeneously expressed in stromal fibroblastic cells, and CD105-positive fibroblasts promote neuroendocrine differentiation of prostate cancer^46^. In addition, in prostate stroma, loss of TGF-β receptor type II expression contributes to tumorigenesis, progression, and invasion^44,47^.

The heterogeneous population of cells in CAFs is further verified with single-cell technology^23^. In the human prostate, six subpopulations of CAFs have been discovered by single-cell RNA sequencing^48^. These CAF clusters secrete a series of different cytokines with variable immunomodulatory property. Among these clusters, one with CCL2 expression aids in the recruitment of myeloid cells to the TME and correlates with poor clinical outcomes or metastatic potential, while another cluster with CXCL-12 expression recruits mast cells, eosinophils, innate lymphoid cells, and T helper 2 cells. Those inflammatory cells contribute to tumor growth and progression through promoting tumor-associated macrophage activation^48^.

The heterogeneity of CAFs is also determined by the coevolution of epithelial and stromal cells and their relationship in each stage of tumor development^23^. CAFs obtained from prostate cancers at different stages revealed that CAF secretome evolves during prostate cancer progression^49^. The molecular profile of growth factors is expressed differentially at different cancer stages^50^. For example, the level of fibroblast growth factor 7 is high in localized prostate cancer-derived CAFs, while matrix metallopeptidase 11 and heat-shock 70 kDa protein 1 A are high in CAFs of metastatic tumors^51^.

Heterogeneity of CAFs has also been studied with the spatial transcriptomics, which revealed the gradients of gene expression in CAFs of multifocal prostate cancers^52^. In the center of tumors, the gene profile of stromal cells is related to the alteration of metabolism and oxidative stress, implying the central tumor growth relies on the energy released by the stroma^52^. Meanwhile, the gene profile of stromal cells in the periphery of tumor is primarily related to inflammation, a stimulator of tumor growth^52–54^.

In addition, in a CAF-rich tumor environment, the balance disruption between extracellular matrix proteases and proteases inhibitors may lead to more aggressive tumors, compared with a CAF-poor environment^23^. This matrix disruption can cause the release of growth factors/cytokines, such as fibroblast growth factor, hepatocyte growth factor, and TGF-β, which modulate cell proliferation, tumor invasion, and immune responses^55,56^.

Overall, the studies suggest that stromal/CAF heterogeneity in prostate TME explains the multiple functions of CAFs in the tumorigenesis. This heterogeneity over the different stages of tumor progression provides further evidence for the theory of tumor-stroma coevolution^57^.

### Hypoxia-driven heterogeneity

In prostate cancer, vessel morphology has often been described as aberrant or disorganized^58^. Microvascular density can vary and higher microvascular density correlates with the metastasis, aggressive phenotype, and stage of the disease^59^. The disorganized vascular network results in spatial and temporal heterogeneity of tumor hypoxia^60^. Using oxygen electrodes to measure hypoxia at different locations of the tumor revealed that the oxygen status changes across tumor foci and even within the same tumor^61^. Hypoxia gene expression signature provides more reliable predictions of hypoxia-dependent changes in TME^62^.

Hypoxia has been studied extensively in the diversity of the tumor phenotype. Hypoxic cells are subjected to selective pressure, with the hostile growth conditions, the most aggressive cells survive and drive tumor growth^63^. Moreover, hypoxia-HIF signaling can lead to mesenchymal-epithelial transition^64^ and promote mesenchymal reprogramming and neuroendocrine transdifferentiation in prostate cancer cells^31^. Low oxygen pressure and poor nutrient supply may result in histone hypermethylation, which induces the tumor epigenetic heterogeneity^65^ and reduces the expression of repressor element 1-silencing transcription (REST), an important epigenetic regulator. The REST in turn leads to neuroendocrine transdifferentiation^66^. The adaptation under hypoxia of cancer cells allows for the survival and proliferation of cancer stem cells^67^, and the emergence of therapy-resistant phenotypes^68^. The cancer stem cells in the TME mediate tumor initiation, metastasis, and therapy resistance^69^. In prostate cancer, four core pluripotency transcription factors, OCT4, SOX2, NANOG, and KLF4, are colocalized and required to maintain pluripotency and self-renewal of embryonic stem cells^70^. Hypoxia can induce the tumor phenotypic diversity by regulating the four factors^71^. Other studies revealed that hypoxia can also induce the electron leakage and the generation of reactive oxygen species, which subsequently cause DNA damage and further develop mutator phenotypes, such as reduced DNA repair, increased mutation rate, genomic instability, and increased metastatic capacity^72,73^.

Taken together, studies on tumor hypoxia heterogeneity suggest that a single tissue biopsy may not provide an accurate global hypoxic status and that biopsies of multiple regions/different time points may be needed to assess spatial and temporal heterogeneity and correctly stratify patients in this context.

### Immunity-driven heterogeneity

The immune system collectively functions to recognize and defend against harmful substances. Both innate and adaptive immune systems have protumor and antitumor effects. Immune cell recruitment and localization in the TME vary greatly across lesions, thus creating spatial heterogeneity. In prostate cancer, immune cells infiltrating the tumor have different functions and compositions in different stages. It has been found that infiltration of CD4 + T cells is involved in prostate cancer progression and metastasis^74^ and neutrophils are associated with poor prognosis^75^, while invariant natural killer (NK) T cells delay prostate cancer progression by crosstalk with tumor-associated macrophages^76^. Also, mast cells induce more serious resistance to chemotherapy and radiotherapy in prostate cancer metastasis patients^77^, and the infiltration of CD8 + T cells in invasive margins is related to poor clinical outcomes^78^. Hence, the frequency and location of immune cells in prostate tissue might be associated with the subclonal heterogeneity of prostate cancer.

The TME of prostate cancer has been described as an immunosuppressive state. This immunosuppressive state can be characterized by poor cytolytic activity of NK cells, which may be contributed through increased TGF-β secretion by prostate tissue, and reduced antitumor immunity from activated T regulatory cells^79^.

This tumor immune complexity and heterogeneity is regulated by a series of factors, such as the secretion of CAFs, the extent and permeability of vasculature, and the tumor cells themselves^80^. Meanwhile, tumor-associated macrophages are well known for their heterogeneity and plasticity, which generally polarize toward two extremes, M1(antitumorigenesis) phenotype and M2 (protumorigenesis) phenotype^81^, and this heterogeneous macrophage activation is often expressed simultaneously in the TME of prostate cancer^82–84^. Typically, M1 macrophage activation is associated with the generation of reactive oxygen species, increased expression of IL-12, and reduced expression of IL-10. In contrast, the M2 phenotypic macrophage acquires the ability to execute protumorigenic and proangiogenic functions through the expression of IL-4, IL-13, and IL-10 cytokines^85^.

Growth differentiation factor 15 (GDF‐15), also known as nonsteroidal anti-inflammatory drug-inducible gene (NAG)-1 or macrophage inhibitory cytokine (MIC)-1, is a member of the TGF-β superfamily proteins. GDF-15 is induced by cellular stress conditions and responds to microenvironment stimulators^86,87^. GDF-15 is dysregulated not only in epithelial cancer cells, but also in the tumor stroma^88^, and highly expressed GDF-15 is associated with worse clinical outcomes in prostate cancer^89,90^. GDF-15 also exhibits the heterogenous functions of either tumor-suppressing or tumor-promoting effects^91,92^. In a landscape of TME, GDF-15 acts on both nonimmune cells and immune cells. For instance, GDF-15 acts on epithelial cancer cells and promotes epithelial-mesenchymal transition through the MAPK/PI3K pathway^93^. GDF-15 also promotes metastasis by activating the SMAD2 and SMAD3 signaling. In addition, with the condition of cellular stress, GDF-15 and other cytokines produced by fibroblasts, such as granulocyte-macrophage colony stimulating factor, interleukin-12 (IL-12), or chemokine c-x-c motif 12 (CXCL-12), act on nearby cells to promote tumor cell proliferation and tumor growth^94^. On the other hand, GDF-15 also acts on immune cells and promotes cancer progression by immune escape. For example, tumor-derived GDF-15 suppresses the proapoptotic activity through inhibiting TGF-β-activated kinase (TAK1) signaling to nuclear factor-κB, thereby evading macrophage-mediated immune surveillance and stimulating early cancer development^86^. GDF-15 preferentially inhibits M1 macrophage, thereby promoting protumorigenic activities^92^. GDF-15 also alters NK cell function by inhibiting TNF-alpha and interferon-gamma production and facilitates the escape of tumor cells from the immune system^95^. Furthermore, GDF-15 promotes the expression of PD-L1 through SMAD2 and SMAD3 signaling, thereby suppressing the activation of T cells through a programmed cell death-1 (PD-1)/PD-L1 interaction^96^. In general, the function of GDF-15 is complex in the TME and uncovering the pathways of GDF-15 in the tumorigenic process would lead to the development of novel therapeutics.

In addition, tumor hypoxia is essential in regulating phenotypic variations of tumor-associated macrophages. Factors released by tumor-associated macrophages play a role in processes such as tumor development, cancer immunosuppression, and angiogenesis^97–99^. Since tumor-associated macrophages are dependent on microenvironmental signals like hypoxia and cytokine availability, it is reasonable to suggest that phenotypic changes occur in a spatiotemporal manner^85^. There are even more signaling pathways that participate in macrophage function and activation, such as tumor necrosis factor, nuclear factor-κB or Toll-like receptor, which are regulated differentially in different tumor foci and further contribute to the phenotypic diversity in the tumor^82^.

Apart from controlling the phenotypic diversity of tumor-associated macrophages, hypoxia also promotes immune escape by upregulating immune checkpoint proteins in tumor foci^100^. Accumulated studies confirm that PD-L1 binds to the PD-1 on the T cells to inhibit the immune response through induction of apoptosis and anergy in the T cells^101^. Hypoxia potently induces HIF-1α-dependent PD-L1 expression on tumor cells^102^, suggesting that PD-L1 expression can be upregulated in hypoxic tumor cells and promote immune escape from cytotoxic T cells. Restoration of T cell infiltration through inhibition of the hypoxia signaling pathway allows prostate cancer to become more susceptible to immunotherapy^103^. These studies suggest that coblockade of PD-L1 and HIF-1α signaling might represent a promising approach to reinforce the activity of cytotoxic T cells.

An important feature of prostate cancer is the low tumor mutation burden and thus diminished neoantigen expression compared with many other cancers^104^. Only 10–30% of unselected patients respond to PD-1/PD-L1 blockade^105^. However, a recent study investigated 1551 prostate tumors and found that 32 (3.1%) prostate cancer specimens were associated with MSI-H molecular phenotype and high tumor mutation burden. More than one-half of the patients who underwent anti-PD-1/PD-L1 therapy had a significant reduction in prostate-specific antigen levels or radiographic responses. In the multifocal sequencing analysis of six patients with MSI-H prostate cancer, the study found that two patients had an acquired MSI-H phenotype in tumors collected further down in the disease progression. Therefore, the findings provide some evidence that MSI-H molecular phenotype can be somatically acquired^106^. It has been shown that in colorectal cancer, MSI-H tumors exhibit greater immune cell infiltration and upregulated immune-related gene expressions, and are more immunogenic than MSI-L tumors^107^. Therefore, it is possible that in prostate cancer, the immune microenvironment can have dynamic changes over time and clinical states and may be altered with therapy^108^. For example, following androgen deprivation therapy, there is an increase in tumor-infiltrating lymphocytes in the prostate bed^109^ and increased PD-L1 expression levels of enzalutamide-treated prostate cancer cells^110^. Preclinical studies have also reported that the Fas expression and antigen presentation can be induced by docetaxel in the TME of prostate cancer^111^.

Taken together, the immune environment of prostate cancer is complex, heterogeneous, and dynamic during disease evolution. It can be influenced by tumor cells, oxidative stress, TME, and anticancer therapies. Exploration of immunity-mediated tumor heterogeneity may help develop precision medicine for prostate cancer.

### Therapy-induced heterogeneity

**Fig. 3:**
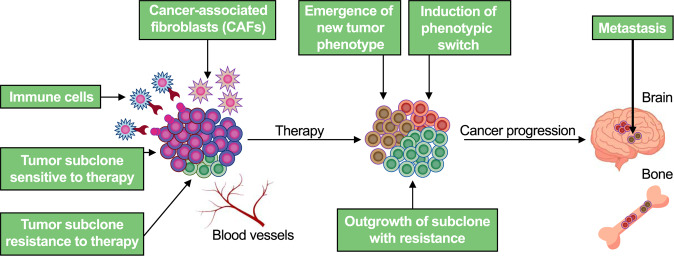
Therapy-mediated tumor heterogeneity. Primary tumors are composed of different subclones. Some subclones are sensitive to selective pressures, including chemotherapy, androgen deprivation therapy, or immunotherapy, while some subclones are resistant to anticancer therapies. Outgrowth of resistant subclones, phenotype switch, or emergence of a new tumor phenotype are strongly related for resistance to anticancer therapies. As a result, the tumor heterogeneity plays a fundamental role in cancer progression to metastasis.

Most advanced metastatic cancers remain incurable, even in those cases that the approaches of therapy are available to eliminate the majority of tumor cells. Pre-existing tumor heterogeneity increases the odds of at least some tumor cells surviving therapy-induced elimination, while ongoing diversification during treatment enables cancer cells to adapt to therapy-imposed selective pressures and facilitates development of new therapy-resistant phenotypes (Fig. 3). Clinical studies have shown that emergence of the polyploid giant cancer cells (PGCCs) phenotype could be a survival strategy for the prostate cancer population and correlates with poor response to docetaxel chemotherapy in the context of castration-resistant prostate cancer^112^. A large number of PGCCs can be induced at higher levels of docetaxel concentration and PGCCs exhibit increased expression of the mesenchymal marker, ZEB1, suggesting that these cells could be a possible inducer of an epithelial-mesenchymal transition and mediators of resistance in response to chemotherapeutic stress^112,113^.

Metastatic castration-resistant prostate cancer (mCRPC) is a heterogeneous disease entity with widely varying outcomes^114,115^. Small cell carcinoma of the prostate is a rare entity and represents one of the most aggressive subsets of mCRPC^116–119^. Analysis of the whole genome and transcriptome of several mCRPC tumor biopsies from the same individual demonstrated that there is a spatial and temporal intrapatient heterogeneity of the metastatic tumors harboring adenocarcinoma, neuroendocrine carcinoma, or mixed expression phenotypes^120^. In the subset of mCRPC tumors, there is a phenotypic switch from an androgen receptor signaling enhanced mCRPC to an androgen receptor signaling reduced small cell carcinoma after androgen deprivation therapy. Owing to few genomic aberrations in neuroendocrine prostate cancer, it has been suggested that the neuroendocrine differentiation could be largely driven by epigenetic dysregulation or signals from the TME^31^. Hence, epigenetic therapy to block neuroendocrine differentiation or reverse the lineage switch and restore sensitivity to androgen deprivation therapy is a promising avenue to improve anticancer therapies, and dual targeting of adenocarcinoma and neuroendocrine carcinoma phenotypes may be needed. In a more recent study, five distinct mCRPC phenotypes were identified after androgen deprivation therapy based on the expression of well-characterized androgen receptor or neuroendocrine genes, including androgen receptor-high tumors, androgen receptor-low tumors, amphicrine tumors composed of cells coexpressing androgen receptor and neuroendocrine genes, double-negative tumors, and tumors with small cell or neuroendocrine gene expression without androgen receptor activity. It was also found that a subtype of mCRPC exhibits features of squamous cell carcinoma^121^. Accumulated evidence also demonstrated that therapy-mediated neuroendocrine differentiation can be induced not only by androgen deprivation therapy and chemotherapy, but also by radiotherapy^122^. Altogether, these studies provide further evidence that tumor heterogeneity can be induced by anticancer therapies and potentially guide future therapeutic studies and clinical trial design^121^.

Approximately 20% of individuals with advanced prostate cancer have somatic or germline mutations in DNA damage repair regulatory genes^104^. In prostate cancer patients with DNA repair mutations, poly-ADP-ribose polymerase (PARP) inhibitors have demonstrated promising effectiveness. Two PARP inhibitors, olaparib and rucaparib, have recently received FDA approval to treat patients with mCRPC harboring BRCA mutations^123^. However, the acquired PARP inhibitor resistance phenotype is an emerging clinical problem. It is crucial to understand resistance mechanisms, which will help formulate subsequent treatment strategies. Extensive preclinical studies have identified several resistance mechanisms for PARP inhibitors^124^. The best-documented mechanism of resistance in prostate cancer is acquired *BRCA2* reversion mutations, where previously *BRCA2*-deficient tumor cells can achieve *BRCA2* proficiency due to constant selective pressure of PARP inhibition. Analysis of circulating cell-free DNA revealed that multiclonal heterogeneity of *BRCA2* reversion mutations plays a key role in resistance to PARP inhibitors^125^. Other inherent or acquired mechanisms to PARP inhibitor resistance include overexpression of drug-efflux transporter genes and multidrug-resistant genes and loss of the *TP53*-binding protein 1 and shielding factors that reactivate homologous recombination or alter intracellular drug concentrations^126^. Analysis of acquired PARP inhibitor-resistant tumor cells showed that some clones present with multiple mechanisms of resistance at the same time^127^. Moreover, PARP inhibitors may also contribute to heterogeneity of the TME or immune environment^128^. Hence, resistance to PARP inhibitors is an immediate result of tumor cell heterogeneity and ongoing diversification during therapy, which induce the emergence of a new tumor phenotype. The optimal way of targeting the heterogeneous PARP inhibitor-resistant clones in combinations or sequentially, should be addressed in the future.

Immunotherapies targeted T cells using engineered T lymphocytes expressing tumor-directed chimeric antigen receptor (CAR) have been designed to treat patients with various malignancies, such as neuroblastoma, B-cell lymphoma, colorectal cancer, and hepatocellular carcinoma^129–132^. However, clinical studies that investigated treating prostate cancer using this technology are still disappointing. For example, the Muc1 expression has been correlated with poor prognosis and an increased risk of disease recurrence^133^. A study found that the anti-Muc1 CAR T cells can selectively kill prostate cancer cells which expressed Muc1. However, due to heterogeneous and fluctuated expression of the Muc1 antigen on prostate cancer cells, the immunotherapy could also induce the outgrowth of target-antigen loss variant or emergence of a new tumor phenotype, thereby leading to immune escape^134^. Expression of prostate-specific membrane antigen (PSMA) and prostate stem cell antigen (PSCA) correlates with clinical stage, invasion, and metastasis in prostate cancer^135^. Both PSMA-targeting and PSCA-targeting CAR T cells have been constructed and tested in the preclinical models. Similar to anti-Muc1 CAR T cells, intratumoral application of anti-PSMA or anti-PSCA CAR T cells kills prostate cancer cells initially, but relapse was observed owing to immune escape^135^.

Taken together, under therapeutic selective pressure, resistance to anticancer therapies can emerge as a result of outgrowth of preexisting resistant subclones or from the evolution of a new tumor phenotype. Cotargeting the drug-sensitive subclones together with the various drug-resistant subclones and newly emerging drug-tolerant tumor cells has been explored as a strategy to induce the most durable responses.

### Challenges of stroma-targeting strategies

Most conventional anticancer therapies and immunotherapy specifically target prostate cancer cells, but the TME can promote the resistance of cancer cells to these therapies. Therefore, targeting TME is an attractive strategy for the treatment of prostate cancer.

Based on the current understanding of tumor heterogeneity, drugs targeting the TME components are under development^136^. However, there are still significant challenges to implement strategies targeting stroma in clinical practice.

Since the complexities of tumor stroma at different stages of prostate cancer development are largely unknown, the first issue is determining the composition of the stroma and accurately modeling tumor stroma complexity and heterogeneity in a preclinical setting^137^. Modeling various nontumor cell types is particularly difficult because current preclinical approaches rely primarily on the implantation of tumor cells in foreign locations, where the stromal representation may differ, or in immunocompromised hosts that lack critical immune effector cells. As a result, the inclusion of different preclinical systems and the development of advanced genetic models are necessary to unravel the components of the TME further and preclinically evaluate the efficacy of combination treatments that target multiple tumor components.

In addition, developing reliable diagnostic markers that target the tumor stroma is also challenging due to the lack of clear understanding of the determinants of responsiveness. For example, despite several attempts to find prognostic biomarkers for castration-resistant prostate cancer, there are currently no confirmed predictive biomarkers available to guide therapeutic decision-making^138^. Tumor heterogeneity may be the reason that biomarkers for treatments targeting the tumor stroma, in general, have remained elusive.

Furthermore, the tumor’s complexity results from ongoing interaction between tumor cells and the environment throughout the course of disease progression, which adds to the challenge of spatiotemporal heterogeneity. Since cancer patients are often treated with multiple lines of therapy, serial biopsies from various locations are necessary to predict treatment responsiveness and make therapeutic decisions.

## Conclusions

Heterogeneity of the TME plays a key role in prostate cancer progression. The currently available therapies for prostate cancers, including conventional therapies and immunotherapy, could lead to therapy-induced tumor heterogeneity. Further characterization of genetic and nongenetic heterogeneity will aid in the development of more effective, personalized, and targeting-specific therapies for advanced prostate cancer patients.

## Data Availability

Any display item and related data are available upon request.
